# Echocardiographic Red Flags in Wild-Type Transthyretin Amyloidosis: Sex-Specific Gaps for Wall Thickness and Left Ventricular Mass

**DOI:** 10.3390/life16020237

**Published:** 2026-02-01

**Authors:** Emilio Nardi, Carola Maria Gagliardo, Davide Noto, Carlo Maria Barbagallo, Antonina Giammanco, Gianluca Di Rosa, Federica Bellini, Maurizio Averna, Angelo Baldassare Cefalù

**Affiliations:** 1 Department of Health Promotion, Mother and Child Care, Internal Medicine and Medical Specialties “G. D’Alessandro” (PROMISE), University of Palermo, 90127 Palermo, Italymaurizio.averna@unipa.it (M.A.); 2 Institute of Biophysics (IBF), National Research Council (CNR), 90125 Palermo, Italy

**Keywords:** cardiac amyloidosis, wild-type transthyretin amyloidosis, echocardiography, red flags, interventricular septal wall thickness, left ventricular hypertrophy, left ventricular mass

## Abstract

**Background:** Wild-type transthyretin amyloidosis (ATTRwt) diagnosis remains challenging. Echocardiographic “red flags” play a significant role in raising diagnostic suspicion. **Methods:** Retrospective study including 33 patients diagnosed with ATTRwt. All patients underwent comprehensive echocardiographic evaluation focusing on the red flags for ATTRwt. Left ventricular hypertrophy (LVH) was defined as interventricular septal wall thickness (IVST) ≥ 12 mm and/or LV mass indexed for body surface area (LVMI) ≥ 115 g/m^2^ in men and ≥ 95 g/m^2^ in women. **Results:** Relative wall thickness > 0.42 and early diastolic myocardial velocity < 7 cm/s were detected in 100% of patients. Severe diastolic dysfunction (grade ≥ 3) (72.7%), apical sparing (36.4%), granular sparkling pattern (30.3%), and pericardial effusion (39.4%) were also observed. Females were younger than males (median age 68 vs. 74.5 years), and IVST ≥ 12 mm was lower in females than in males (64.4% vs. 100%, respectively, *p* < 0.05). The combined criterion of IVST ≥ 12 mm in men and LVMI ≥ 95 g/m^2^ in women was encountered in 100% of the global cohort. **Conclusions:** IVST is a good predictor of LVH in males but shows limited sensitivity for ATTRwt in females; a gender-differenced approach (IVST for men and LVMI for women) might better stratify for ATTRwt suspicion.

## 1. Introduction

Cardiac amyloidosis (CA) is a progressive infiltrative cardiomyopathy characterized by the extracellular deposition of misfolded protein fibrils within the myocardium. The two most common forms are light chain (AL) amyloidosis and transthyretin amyloidosis (ATTR), which encompasses both wild-type (ATTRwt) and hereditary variants (ATTRv) [1,2].

In recent years, the implementation of a simplified and less invasive diagnostic algorithm has facilitated early diagnosis and revealed that this condition should be considered less rare than previously thought [3]. Advanced imaging technologies such as echocardiography, cardiac magnetic resonance imaging (CMR), and bone scintigraphy play a pivotal role in identifying structural and functional abnormalities associated with CA [4].

Beyond patient history, clinical signs, and electrocardiographic features, the diagnostic suspicion of ATTRwt is most frequently raised by echocardiographic ‘red flags’: increased left ventricular (LV) wall thickness (LVWT), “Granular sparkling” appearance of the myocardium, preserved or mildly reduced left ventricular ejection fraction (LVEF) with diastolic dysfunction, right ventricular wall thickening (RVWT), valvular thickening (aortic stenosis is frequently observed), pericardial effusion, and reduction in left ventricular global longitudinal strain (GLS).

One of the hallmark features of ATTRwt is left ventricular hypertrophy (LVH), which is not due to a real myocyte hypertrophy, as in hypertensive or hypertrophic cardiomyopathy, but rather due to amyloid infiltration of the myocardial interstitium. This pseudo-hypertrophy is often paradoxically disproportionate to the low voltage seen on electrocardiography (ECG)—a typical finding that should raise suspicion for amyloidosis [3,5]—and to the degree of diastolic dysfunction. In particular, it is not uncommon to observe a mild increase in wall thickness associated with severe diastolic dysfunction of the left ventricle.

LVH in cardiac amyloidosis is typically concentric and may be accompanied also by bi-atrial enlargement, thickening of the atrioventricular valves, and interatrial septum. Notably, “Apical Sparing” (AS) pattern at GLS analysis is another sensitive echocardiographic marker for CA [6].

The presence of LVH in the absence of hypertension or valvular disease should prompt evaluation for CA, especially in patients presenting with heart failure with preserved EF (HFpEF), arrhythmias, or conduction abnormalities. Tissue biopsy remains the gold standard for diagnosis, although non-invasive techniques are increasingly reliable for subtype differentiation [7].

Given the key role of echocardiography in supporting the diagnostic suspicion of CA, several studies have been conducted to develop specific scoring systems that may be useful for this purpose. Major criteria usually include an unexplained LVWT or interventricular septal thickness (IVST) ≥ 12 mm, associated with at least one additional typical echocardiographic feature [7,8,9].

In this retrospective study, we focused on the echocardiographic red flags, emphasizing LVH-related parameters and LVWT in patients diagnosed with ATTRwt.

## 2. Materials and Methods

This is a retrospective study conducted on patients admitted either to the Emergency Medicine Unit or evaluated as outpatients at the Rare Metabolic Diseases Unit of the University Hospital ‘Paolo Giaccone′ in Palermo, from 2022 to the end of 2025. The study included a total of 33 patients with confirmed ATTRwt diagnosis.

Patients with hemodynamically significant aortic stenosis, hematological malignancies, uncontrolled blood pressure and other cardiomyopathies were excluded.

According to the most recent position statements, patients with clinical and echocardiographic red flags of CA underwent biochemical examinations to rule out AL amyloidosis [serum free light chain (FLC) assay, serum and urine protein electrophoresis (SPIE and UPIE) with immunofixation] and bone scintigraphy with 99mTc-pyrophosphate, applying Perugini scoring system (Grade 2: similar myocardial and bone uptake and Grade 3: myocardial uptake greater than bone with reduced/absent bone uptake) [7].

In one doubtful case, a myocardial biopsy was needed to confirm diagnosis; genetic tests were performed [7].

All patients underwent routine 12-lead electrocardiography and comprehensive transthoracic echocardiography, including color Doppler, tissue Doppler, and speckle-tracking analysis.

Echocardiographic assessment was performed using a Canon Aplio i700 ultrasound system, with off-line analysis on a Vitrea workstation (Canon), as previous studies [10].

Echocardiographic parameters were evaluated according to the guidelines of the American Society of Echocardiography (ASE) and are listed in Table 1 [11,12].

In addition, NYHA classes and clinical heart failure (HF) phenotype were also evaluated [13]. HF phenotype was defined according to LVEF%: HF with reduced ejection fraction (HFrEF, LVEF ≤ 40%), HF with mildly reduced ejection fraction (HFmrEF, LVEF 41–49%), and HF with preserved ejection fraction (HFpEF, LVEF ≥ 50%, and evidence of cardiac structural and/or functional abnormalities consistent with the presence of LV diastolic dysfunction/raised LV filling pressures).

LVM was determined using the ASE-corrected cube formula [13] and was indexed (LVMI) by body surface area (BSA—m^2^). LVH was defined as IVST ≥ 12 mm and/or LVMI ≥ 115 g/m^2^ in men and ≥ 95 g/m^2^ in women, according to the 2018 ESH/ESC guidelines [14]. RWT was calculated as the 2PWT/LVEDD ratio. LVEF was assessed by the biplane modified Simpson method [14]. Diastolic function was evaluated using mitral inflow and tissue Doppler echocardiography, according to ASE recommendations [15].

Mitral inflow was assessed in the apical four-chamber view, using pulsed wave Doppler echocardiography, with the Doppler beam aligned parallel to the direction of flow and the sample volume at the leaflet tips. From the mitral inflow profile, the E-wave (E) and A-wave (A) peak velocities, E/A ratio, and deceleration time of E-wave (DT-E) were measured. Tissue Doppler imaging of the mitral annulus was obtained from the apical four-chamber view using a 1–2 mm sample volume placed in the lateral mitral valve annulus to evaluate early diastolic myocardial velocity (e′) [16].

In addition, IAS and ML thickness were measured, applying a 5 mm cut-off.

GLS and AS patterns (calculated as the apical-to-basal ratio of systolic longitudinal strain > 2.9) were assessed by 2D speckle-tracking [5].

Additionally, a combined gender-differenced echocardiographic criterion (IVST ≥ 12 mm for men and an LVMI > 95 g/m^2^ for women) was explored.

After image acquisition, all images were stored in DICOM format on a hard disk. A random sample of 10 recorded exams was selected to evaluate the variability within and between observers. Two experienced ultrasound physicians independently evaluated the two-dimensional images and measures.

Following a 2-week interval, the same physician reanalyzed the recorded echocardiograms of the selected subjects. Intra-observer variability was assessed by comparing the measurements from the first physician’s two assessments, while inter-observer variability was evaluated based on the results from both physicians. The intra-observer and inter-observer correlation coefficients were considered excellent. The intra-observer and inter-observer variabilities were higher than 0.8.

All patients’ data were anonymized. Since the retrospective nature of the study, according to our Institutional Ethic Committee and Italian national regulations, ethical approval was not required. However, each patient was informed regarding data collection and gave their consent to data publication in anonymized form.

### Statistics

Descriptive statistical analysis reported medians and 95% confidence interval (CI), as the data did not meet the normality assumptions. The Mann–Whitney U test and Fisher exact tests (non-parametric tests) were applied for numerical and categorical variables, respectively, to analyze differences between male and female patients. Alpha error was set ≤0.05 for significancy. Effect size “r” was calculated based on Z value using the Mann–Whitney U test: r = Z/√N. Cramer’s V effect size (Φ) was calculated for Fisher exact test.

All the calculations were performed by the R scientific software (version 4.3.2) running within Rstudio suite graphical interface.

## 3. Results

The study included a total of 33 patients: 19 males and 14 females. Table 2 summarizes the characteristics of the patients included in the study. Median age was 73 years, and females were younger than males (median age 68 years vs. 74.5, respectively, *p =* 0.02). BSA was also lower in females (median 1.6 vs. 1.78, respectively, *p* = 0.0001).

None of the patients were classified as NYHA I; A total of 27.3% were classified as NYHA II, 48.5% as NYHA III, and the remaining 24.2% as NYHA IV, without significant differences between males and females.

At echocardiographic evaluation, 5 out of 33 patients (15.2%) showed reduced LVEF%, 10 out of 33 patients (30.3%) mildly reduced, and 18 out of 33 patients (54.5%) preserved LVEF%. By sub-phenotyping heart failure according to LVEF and gender, male patients exhibited a greater prevalence of HFmrEF than females (52.6 vs. 0%, respectively, *p* = 0.001); female patients conversely showed a higher prevalence of HFrEF (28.6 vs. 5.3%, respectively). However, LVEF% values were globally lower in the male group, as evidenced by a higher prevalence of LVEF% < 50% (57.9% vs. 28.6% in females) and lower LVEF% median values (45 vs. 52 in females), although data not encountered statistical significancy.

Five patients (all females) out of thirty-three had an IVST measurement < 12 mm cut-off, which is generally considered a ‘red flag’ for diagnosis. None of the patients had an IVST/PWT ratio greater than 1.3.

Median LVMI values were higher in the female group (148.39 gr/m^2^) than in males (128.42 gr/m^2^), despite not statistically significant.

Median RWT value was 0.58, with a median value higher in male (0.64) than female patients (0.56). No patient had an RWT value lower than 0.42, which is considered the cut-off for concentric hypertrophy (12); the cut-off ≥ 0.57 was exceeded in 57.6% of the study population. No patient had a dilated left ventricle, as evidenced by the median LVEDD values (Table 1).

Only 3 out of the 33 patients had a normal-sized left atrium (<20 cm^2^).

A pericardial effusion was detected in 39.4% of the patients, and it was more prevalent in males (47.4% vs. 28.6%).

No patient exhibited normal transmitral diastolic function: 60.6% had a restrictive pattern (grade 3), 24.2% a pseudonormal pattern (grade 2), and 15.2% impaired relaxation (grade 1). Globally, 72.2% of the subjects showed a grade ≥ 3. Diastolic dysfunction was confirmed by very low e′ values on tissue Doppler imaging (minimum registered value: 3 cm/s) and markedly elevated E/e′ ratio (maximum registered value: 43), without significant gender-related differences. The median GLS value was reduced (−15%), more in males. A granular sparkling myocardial pattern was observed in 30.3% of patients, while AS was present in 36.4%. IAS and ML thickening was observed in 45.5% and 54.5% of the cases, respectively.

**Figure 1:**
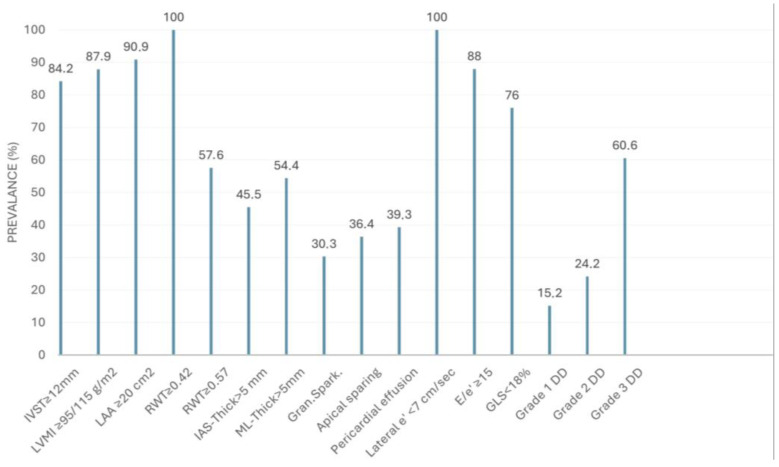
Prevalence of echocardiographic evaluated parameters. IVST: interventricular septum thickness; LAA: left atrium area; LVMI: left ventricular mass indexed for body surface area; RWT: relative wall thickness; GLS: global longitudinal strain; IAS-thickness: interatrial thickness; ML: mitral leaflet thickness. Gran. Spar: granular sparkling; DD: diastolic dysfunction.

Among all echocardiographic parameters, only RWT > 0.42 and lateral e′ < 0.07 m/s reached a prevalence of 100% (see Figure 1).

**Figure 2:**
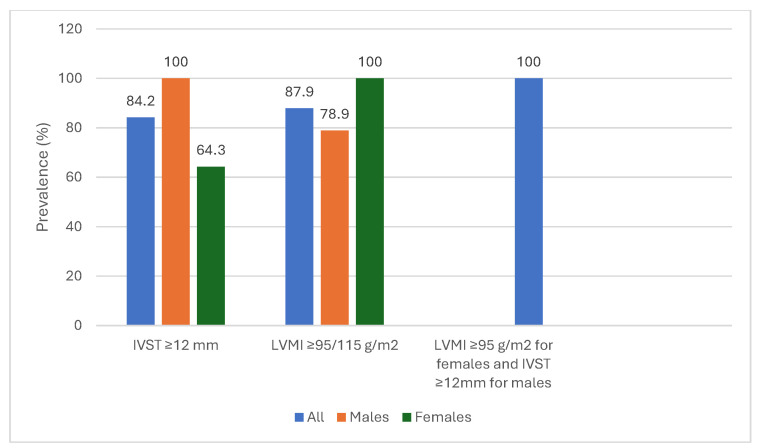
Prevalence of LVMI and IVST stratified by sex.

By considering a differential echocardiographic approach according to sex-differences, the IVST ≥ 12 mm criterion was globally satisfied in 84.8% of the entire cohort, but it was more prevalent in males than females (100% vs. 64.3%, respectively, *p* = 0.0084). LVMI cut-off ≥ 115 g/m^2^ for males and ≥95 g/m^2^ for females was reached by 87.9% of the patients but differentially in the two groups: 78.9% in males vs. 100% in females (see Table 2). The combined gender-differenced echocardiographic criterion (IVST ≥ 12 mm for men and an LVMI ≥ 95 g/m^2^ for women) was satisfied in 100% of the study cohort (see Figure 2).

The graph shows the prevalence (%) for left ventricular mass indexed for body surface area (LVMI) and interventricular septum thickness (IVST), stratified by sex. In the right side of the graphic, blue column alone shows the prevalence of the combined criterion IVST cut-off ≥ 12 mm for males and LVMI cut-off ≥ 95 g/m^2^ for females (encountered in 100% of entire population).

## 4. Discussion

The recent availability of disease-modifying therapies for CA, particularly ATTRwt, has placed increasing emphasis on ensuring early diagnosis to positively influence patient prognosis.

Echocardiography plays a key role for ATTRwt diagnosis since it is non-invasive, easily repeatable, and well tolerated by patients. Unexplained IVST ≥ 12 mm is an echocardiographic criterion of CA, when associated with at least one of the following measures [7,8,9]:

1. At least two echocardiographic features:
  ○ Diastolic dysfunction of grade ≥ II;
  ○ Reduced velocity (<5 cm/s) of s′, e′, and a′ waves on tissue Doppler imaging;
  ○ Reduced global longitudinal strain (GLS) of the LV (absolute value < −15%).
2. Increased wall thickness (IWS) Score ≥ 8 points:
  ○ RWT of the LV > 0.6 (3 points);
  ○ E/e′ > 11 (1 point);
  ○ TAPSE (tricuspid annular plane systolic excursion) ≤ 19 mm (2 points);
  ○ LV GLS ≤ −13% (1 point);
  ○ Apex-to-base ratio of systolic longitudinal strain > 2.9 (3 points).

Another tool to identify ATTR-HFpEF is the ATTR-CM score [17], which includes three clinical parameters (age, male sex, arterial hypertension) and three echocardiographic criteria (LVEF, PWT, and RWT). Patients at high risk for ATTR-HFpEF (score is ≥6) may undergo bone scintigraphy, thus improving ATTRwt identification.

Despite the advance in the use of scoring systems and the improving accuracy in defining ATTR red flags, many subjects still remain undiagnosed or receive the diagnosis late at advanced stage of the disease, when currently available drugs become less effective.

Our study focused on the prevalence of echocardiographic red flags in patients with ATTRwt, with particular emphasis on indices of LVH. We provide both a clinical and echocardiographic evaluation of ATTRwt patients highlighting more advanced stages of HF in females than males.

The more severe disease and the disproportion between lower values of LVWT and high LVMI values in females than in males should prompt to identify other early echocardiographic markers for ATTRwt detection.

In all patients, a severe diastolic dysfunction was observed by the reduced e′ values and the markedly elevated E/e′ ratio. Severe diastolic dysfunction is uncommon in patients with concentric LVH secondary to other conditions (such as arterial hypertension or aortic valve stenosis). Therefore, the degree of the diastolic dysfunction should raise suspicion of CA, particularly in women with disproportional low IVST values.

In fact, IVST ≥ 12 mm was less prevalent among female patients. This finding is consistent with previous reports showing that affected female patients have reduced LVWT and higher LVMI values [18].

It is widely accepted that females have lower IVSWT, PWT, and LVEDD than males in both normal and amyloidotic hearts [19].

Gender differences in ATTRwt patients are debated. Functional and structural echocardiographic profile seems more severe in women than in men (higher concentric hypertrophy and LV filling pressures, worse diastolic and RV systolic functions) [20]. However, cardiovascular and overall mortality seems to be not influenced by gender [21].

To our knowledge, there are no studies that have clarified the pathophysiological mechanism of these differences. It does not seem that gender differences contribute to a differential fibril deposition mechanism, while it is currently thought that estrogens could play a protective role in cardiomyocytes that delay the progression of CA in women, but also leading to a significant delay in diagnosis [22,23].

Recent analyses, as the “*THAOS Survey-Outcomes*” study [23], have shown that sex-related differences are markedly attenuated when myocardial wall thickness is normalized to BSA, indicating that cardiac involvement is only apparent less severe in women. The evaluation alone of IVST in women, without considering LVMI or BSA, has contributed to a greater diagnostic delay in women, with a higher proportion of post-mortem diagnoses [24]. These considerations are consistent with our results, which demonstrated lower IVST values but LVMI ≥ 95 g/m^2^ in all females (Figure 2).

More, the elevated RWT values were also expected in patients with concentric LVH. Other parameters, such as granular sparkling and apical sparing, although more specific than other parameters, showed a relatively low prevalence. The reduced median GLS values may have limited diagnostic utility, unless extremely low values are observed.

The take-home message of this manuscript could be the use of a gender-differenced echocardiographic criterion to suspect ATTRwt diagnosis: IVST cut-off ≥ 12 mm for men and an LVMI cut-off ≥ 95 g/m^2^ for women. This combined criterion needs further validation in larger cohorts in order to be considered for the ATTRwt diagnostic work-up.

**Limits of the study:** This is a pilot retrospective study. The lack of a control group and the small sample size of the cohort limit the statistical power of our analyses.

## 5. Conclusions

In conclusion, our findings offer useful insights into echocardiographic assessment of ATTRwt. Given the limited sensitivity of IVST values in women, a gender-differenced echocardiographic approach might be more reliable to suspect ATTRwt diagnosis. Future studies with larger cohorts are needed to validate these observations and refine sex-specific diagnostic criteria.

## Figures and Tables

**Table 1:** Echocardiographic parameters evaluated in all patients according to the American Society of Echocardiography (ASE) guidelines.

LV end-diastolic diameter (LVEDD)
IVST and posterior wall thickness (PWT)
LV mass indexed to body surface area (LVMI)
Relative wall thickness (RWT)
LV ejection fraction (LVEF%)
Left atrial area (LAA)
‘Granular sparkling’ evaluation
GLS by speckle-tracking analysis
AS pattern on speckle-tracking evaluation
Interatrial septal (IAS) thickness
Mitral leaflet (ML) thickness
Presence of pericardial effusion
E-wave velocity on transmitral Doppler flow
Diastolic dysfunction grade by transmitral Doppler flow analysis
Tissue Doppler lateral e′ velocity and E/e′ ratio.

**Table 2:** Clinical and echocardiographic parameters of the ATTR study cohort.

Numeric Variables	Total N = 33	Males N = 19	Females N = 14	*p*-Value *	Effect Size (r) *
	**Median (95% C.I.)**	**Median (95% C.I.)**	**Median (95% C.I.)**		
Age (years)	73 (57–85)	74 (67–85)	68 (57–77)	**0.020**	0.41
LVEDD (mm)	46 (34–55)	45 (39–55)	48 (34–52)	0.898	−0.03
IVST (mm)	13.5 (11–20)	14 (12–20)	13 (11–17)	0.095	0.29
PWT (mm)	13 (10–17)	13 (11–17)	13 (10–17)	0.486	0.12
BSA (m^2^)	1.72 (1.49–2.1)	1.78 (1.49–2.1)	1.6 (1.53–1.85)	**0.0001**	0.58
LVMI (gr/m^2^)	134.05 (99.38–212.1)	128.42 (100–212.1)	148.39 (99.38–181.05)	0.870	0.03
RWT	0.58 (0.42–1)	0.64 (0.45–0.85)	0.56 (0.42–1)	0.353	0.16
LAA (m^2^)	24 (17–37)	28 (17–37)	22 (18–36)	0.212	0.22
LVEF %	52 (30–60)	45 (30–55)	52 (35–60)	0.203	−0.22
E/A	2 (0.5–3.8)	2.4 (0.5–3.33)	2 (0.64–3.8)	0.841	0.04
Lateral e′ (m/s)	0.04 (0.02–0.07)	0.04 (0.03–0.07)	0.04 (0.02–0.06)	0.491	0.12
E/e′	25 (12–43.33)	25 (12–33.33)	25 (14–43.33)	0.369	−0.16
GLS average %	−15 (−21–−10)	−15 (−19–−10)	−18 (−21–−12)	0.088	0.30
TAPSE (mm)	19 (7–23)	20 (7–23)	18 (8–23)	0.595	0.10
**Categorical** **Variables**	**Total** **N = 33**	**Males** **N = 19**	**Females** **N = 14**	***p*-Value #**	**Effect Size (Φ)**
NYHA 1	0 (0%)	0 (0%)	0 (0%)	-	-
NYHA 2	9 (27.3%)	5 (26.3%)	4 (28.6%)	1.000	0.03
NYHA 3	16 (48.5%)	10 (52.6%)	6 (42.9%)	0.728	0.10
NYHA 4	8 (24.2%)	4 (21.1%)	4 (28.6%)	0.695	0.09
HFrEF	5 (15.2%)	1 (5.3%)	4 (28.6%)	0.138	0.32
HFmrEF	10 (30.3%)	10 (52.6%)	0 (0.0%)	**0.001**	0.57
HFpEF	18 (54.5%)	8 (42.1%)	10 (71.4%)	0.158	0.29
Pericardial effusion	13 (39.4%)	9 (47.4%)	4 (28.6%)	0.310	0.19
Grade 1 D.D.	5 (15.2%)	3 (15.8%)	2 (14.3%)	1.000	0.02
Grade 2 D.D.	8 (24.2%)	4 (21.1%)	4 (28.6%)	0.695	0.09
Grade 3 D.D.	20 (60.6%)	12 (63.2%)	8 (57.1%)	1.000	0.06
Granular Sparkling	10 (30.3%)	5 (26.3%)	5 (35.7%)	0.707	0.10
Apical Sparing	12 (36.4%)	8 (42.1%)	4 (28.6%)	0.486	0.14
IAS-Thickness > 5 mm	15 (45.5%)	8 (42.1%)	7 (50.0%)	0.733	0.08
ML-Thickness > 5 mm	18 (54.5%)	11 (57.9%)	7 (50.0%)	0.733	0.08
LVMI ≥ 115 (M)/≥95 g/m^2^ (F)	29 (87.9%)	15 (78.9%)	14 (100%)	0.119	0.32
IVST ≥ 12 mm	26 (84.8%)	19 (100%)	9 (64.3%)	**0.0084**	0.49

Table featuring the investigated clinical and echocardiographic parameters in the ATTRwt study cohort, subdivided for sex. Numerical data are reported as median and 95% confidence intervals (C.Is), categorical data as prevalence (%). *p*-value *: Mann–Whitney U test and relative effect size (r = Z/√N). *p*-value #: Fisher exact test and relative Cramer’s V effect size (Φ). LVEDD: left ventricular end-diastolic diameter; IVST: interventricular septum thickness; PWT: posterior wall thickness; RWT: relative wall thickness; LAA: left atrial area; BSA: body surface area; LVMI: left ventricular mass indexed for body surface area. LVEF %: left ventricular ejection fraction; HFrEF: heart failure with reduced ejection fraction; HFmrEF: heart failure with mildly reduced ejection fraction; HFpEF: heart failure with preserved ejection fraction; D.D: diastolic dysfunction; GLS: global longitudinal strain; IAS-thickness: interatrial thickness; ML: mitral leaflet thickness. Bold type was used to highlight statistical significancy.

## Data Availability

The original contributions presented in this study are included in the article. Further inquiries can be directed to the corresponding authors.

## Abbreviations

CA	Cardiac Amyloidosis
ATTRwt	Wild-type Transthyretin Cardiac Amyloidosis
LVEDD	Left ventricular end-diastolic diameter
IVST and PWT	Interventricular septal and posterior wall thickness
LVMI	Left ventricular mass indexed for body surface area
BSA	Body surface area
RWT	Relative wall thickness
LVEF	Left ventricular ejection fraction
LAA	Left atrial area
GLS	Global longitudinal sparing
IAS thickness	Interatrial septal thickness
HF	Heart failure
HFrEF	with reduced ejection fraction
HFmrEF	with mildly reduced ejection fraction
HFpEF	with preserved ejection fraction

## References

[1] Griffin J.M., Rosenblum H., Maurer M.S. (2021). Pathophysiology and Therapeutic Approaches to Cardiac Amyloidosis. Circ. Res..

[2] Nguyen D.M., Ramazani N., Sodhi G., Tak T. (2025). Cardiac Amyloidosis: Tribulations and New Frontiers. J. Pers. Med..

[3] Merlo M., Pagura L., Porcari A., Cameli M., Vergaro G., Musumeci B., Biagini E., Canepa M., Crotti L., Imazio M. (2022). Unmasking the Prevalence of Amyloid Cardiomyopathy in the Real World: Results from Phase 2 of the AC-TIVE Study, an Italian Nationwide Survey. Eur. J. Heart Fail..

[4] Maurer M.S., Elliott P., Merlini G., Schwartz J.H., Gundapaneni B., Hahn C., Riley S., Schwartz J., Sultan M.B., Rapezzi C. (2017). Design and Rationale of the Phase 3 ATTR-ACT Clinical Trial (Tafamidis in Transthyretin Cardiomyopathy Clinical Trial). Circ. Heart Fail..

[5] Kittleson M.M., Maurer M.S., Ambardekar A.V., Bullock-Palmer R.P., Chang P.P., Eisen H., Nair A.P., Nativi-Nicolau J., Ruberg F.L., On behalf of the American Heart Association Heart Failure (2020). Cardiac Amyloidosis: Evolving Diagnosis and Management: A Scientific Statement From the American Heart Association. Circulation.

[6] Phelan D., Collier P., Thavendiranathan P., Popović Z.B., Hanna M., Plana J.C., Marwick T.H. (2012). Relative Apical Sparing of Longitudinal Strain using two-dimensional speckle-tracking echocardiography is both sensitive and specific for the diagnosis of cardiac amyloidosis. J. Am. Coll. Cardiol..

[7] Garcia-Pavia P., Rapezzi C., Adler Y., Arad M., Basso C., Brucato A., Burazor I., Caforio A.L.P., Damy T., Eriksson U. (2021). Diagnosis and treatment of cardiac amyloidosis: A position statement of the ESC Working Group on Myocardial and Pericardial Diseases. Eur. Heart J..

[8] Boldrini M., Cappelli F., Chacko L., Restrepo-Cordoba M.A., Lopez-Sainz A., Giannoni A., Aimo A., Baggiano A., Martinez-Naharro A., Whelan C. (2020). Multiparametric Echocardiography Scores for the Diagnosis of Cardiac Amyloidosis. JACC Cardiovasc. Imaging.

[9] Liang S., Liu Z., Li Q., Zhang Y., Zhang W., Du Z., Liu L. (2023). Advance of Echocardiography in Cardiac Amyloidosis. Heart Fail. Rev..

[10] Cefalù A.B., Nardi E., Giammanco A., Gagliardo C.M., Barbagallo C.M., La Grutta L., Toia P., Brucato F., Scrimali C., Fasciana T.M.G. (2025). Echocardiographic Calcium Score of Aortic Valve Correlates with Coronary Artery Calcium Score in Heterozygous Familial Hypercholesterolemia. Life.

[11] Sahn D.J., DeMaria A., Kisslo J., Weyman A. (1978). Recommendations Regarding Quantitation in M-Mode Echocardiography. Circulation.

[12] Lang R.M., Badano L.P., Mor-Avi V., Afilalo J., Armstrong A., Ernande L., Flachskampf F.A., Foster E., Goldstein S.A., Kuznetsova T. (2015). Recommendations for Cardiac Chamber Quantification by Echocardiography in Adults: An Update from the American Society of Echocardiography and the European Association of Cardiovascular Imaging. J. Am. Soc. Echocardiogr..

[13] McDonagh T.A., Metra M., Adamo M., Gardner R.S., Baumbach A., Böhm M., Burri H., Butler J., Čelutkienė J., Chioncel O. (2023). 2023 Focused Update of the 2021 ESC Guidelines for the diagnosis and treatment of acute and chronic heart failure: Developed by the task force for the diagnosis and treatment of acute and chronic heart failure of the European Society of Cardiology (ESC) With the special contribution of the Heart Failure Association (HFA) of the ESC. Eur. Heart J..

[14] Williams B., Mancia G., Spiering W., Agabiti-Rosei E., Azizi M., Burnier M., Clement D.L., Coca A., de Simone G., Dominiczak A. (2018). 2018 ESC/ESH Guidelines for the Management of Arterial Hypertension. Eur. Heart J..

[15] Shan K., Bick R.J., Poindexter B.J., Shimoni S., Letsou G.V., Reardon M.J., Howell J.F., A Zoghbi W., Nagueh S.F. (2000). Relation of tissue Doppler derived myocardial velocities to myocardial structure and beta-adrenergic receptor density in humans. J. Am. Coll. Cardiol..

[16] Rivas-Gotz C., Khoury D.S., Manolios M., Rao L., Kopelen H.A., Nagueh S.F. (2003). Time Interval between Onset of Mitral Inflow and Onset of Early Diastolic Velocity by Tissue Doppler: A Novel Index of Left Ventricular Relaxation: Experimental Studies and Clinical Application. J. Am. Coll. Cardiol..

[17] Davies D.R., Redfield M.M., Scott C.G., Minamisawa M., Grogan M., Dispenzieri A., Chareonthaitawee P., Shah A.M., Shah S.J., Wehbe R.M. (2022). A Simple Score to Identify Increased Risk of Transthyretin Amyloid Cardiomyopathy in Heart Failure With Preserved Ejection Fraction. JAMA Cardiol..

[18] Planté-Bordeneuve V., Suhr O.B., Maurer M.S., White B., Grogan D.R., Coelho T. (2013). The Transthyretin Amyloidosis Outcomes Survey (THAOS) registry: Design and methodology. Curr. Med. Res. Opin..

[19] Ochi Y., Kubo T., Baba Y., Sugiura K., Ueda M., Miyagawa K., Noguchi T., Hirota T., Hamada T., Yamasaki N. (2021). Wild-Type Transthyretin Amyloidosis in Female Patients—Consideration of Sex Differences. Circ. Rep..

[20] Fragner M., Elsaygh J., Srivats S.S., Pink K. (2024). Gender Differences in the Evaluation and Management of New Acute CHF Due to ATTRwt Cardiac Amyloidosis. Cureus.

[21] Zampieri M., Argirò A., Allinovi M., Tassetti L., Zocchi C., Gabriele M., Andrei V., Fumagalli C., Di Mario C., Tomberli A. (2022). Sex-related differences in clinical presentation and all-cause mortality in patients with cardiac transthyretin amyloidosis and light chain amyloidosis. Int. J. Cardiol..

[22] Iorga A., Cunningham C.M., Moazeni S., Ruffenach G., Umar S., Eghbali M. (2017). The protective role of estrogen and estrogen receptors in cardiovascular disease and the controversial use of estrogen therapy. Biol. Sex Differ..

[23] Campbell C.M., LoRusso S., Dispenzieri A., Kristen A.V., Maurer M.S., Rapezzi C., Lairez O., Drachman B., Garcia-Pavia P., Grogan M. (2022). THAOS Investigators. Sex Differences in Wild-Type Transthyretin Amyloidosis: An Analysis from the Transthyretin Amyloidosis Outcomes Survey (THAOS). Cardiol. Ther..

[24] Grogan M., Scott C.G., Kyle R.A., Zeldenrust S.R., Gertz M.A., Lin G., Klarich K.W., Miller W.L., Maleszewski J.J., Dispenzieri A. (2016). Natural History of Wild-Type Transthyretin Cardiac Amyloidosis and Risk Stratification Using a Novel Staging System. J. Am. Coll. Cardiol..

